# The Role of Functional Urban Areas in the Spread of COVID-19 Omicron (Northern Spain)

**DOI:** 10.1007/s11524-023-00720-3

**Published:** 2023-02-24

**Authors:** Olga De Cos, Valentín Castillo, David Cantarero

**Affiliations:** 1 Department of Geography, Urban and Regional Planning, Universidad de Cantabria, 39005 Santander, Spain; 2 Research Group on Health Economics and Health Services Management – Valdecilla Biomedical Research Institute (IDIVAL), 39011 Santander, Spain; 3 Department of Economics, Universidad de Cantabria, 39005 Santander, Spain

**Keywords:** Space-time trend, Emerging hot spots, Functional urban areas, Geographic Information Systems, Municipalities

## Abstract

This study focuses on the space-time patterns of the COVID-19 Omicron wave at a regional scale, using municipal data. We analyze the Basque Country and Cantabria, two adjacent regions in the north of Spain, which between them numbered 491,816 confirmed cases in their 358 municipalities from 15th November 2021 to 31st March 2022. The study seeks to determine the role of functional urban areas (FUAs) in the spread of the Omicron variant of the virus, using ESRI Technology (ArcGIS Pro) and applying intelligence location methods such as 3D-bins and emerging hot spots. Those methods help identify trends and types of problem area, such as hot spots, at municipal level. The results demonstrate that FUAs do not contain an over-concentration of COVID-19 cases, as their location coefficient is under 1.0 in relation to population. Nevertheless, FUAs do have an important role as drivers of spread in the upward curve of the Omicron wave. Significant hot spot patterns are found in 85.0% of FUA area, where 98.9% of FUA cases occur. The distribution of cases shows a spatially stationary linear correlation linked to demographically progressive areas (densely populated, young profile, and with more children per woman) which are well connected by highways and railroads. Based on this research, the proposed GIS methodology can be adapted to other case studies. Considering geo-prevention and WHO Health in All Policies approaches, the research findings reveal spatial patterns that can help policymakers in tackling the pandemic in future waves as society learns to live with the virus.

## Introduction

### Background: Geo-technologies and COVID-19 Spatial Patterns

From the outset of the COVID-19 pandemic, geo-technologies and location intelligence methods [1] have been used to help determine COVID-19 spatial patterns [2–4]. These contributions are aligned with geo-prevention principles to detect COVID-19 problem areas [5] in line with WHO Social Determinants of Health [6], Health in All Policies (HiAP) and Healthy Cities principles [7, 8] to reduce inequity in health conditions or pandemic risks depending on the area (urban or rural) or the domains of Social Determinants of Health regarding to living context (neighborhood and built environment) and socioeconomic context [9]. According to this, health is influenced not only by living and working conditions in homes and communities, but also by economic and social opportunities and resources [10]. However, academic studies encounter many difficulties in terms of administrative mismatching of diagnosis areas and health management units [11]. Local studies are essential for action at local scales, but global thinking is needed [12], considering both worldwide and local research about spatial patterns of the pandemic.

Health Geography contributions highlight that the living environment matters in the spatial behavior of COVID-19, especially in urban areas [13]. There is strong evidence of correlation between the distribution of confirmed cases and population density [14–16], environmental conditions such as pollution [17], socioeconomic conditions [18–20], concentration of economic activities [21, 22], proximity to transport centers [23], and indeed proximity to other locations [24].

The context in which people live is important not only in COVID-19 incidence but also in vaccination levels [25]. Nevertheless, as some authors have hypothesized, vaccination seems insufficient to contain spread [26], so public health policy must meet the challenge of high transmissibility variants such as Omicron one, which can spread globally [27].

### Research Questions and Case Study

The research is approached in the awareness of the importance of tackling the pandemic at local scales, coordinating actions horizontally and vertically from an administrative perspective in line with WHO HiAP principles [8]. In this regard, the Omicron wave has posed a challenge to health policies due to its high transmission rates. We seek to analyze the role of urban areas in the spread of the COVID-19 Omicron variant, using geo-statistics methods implemented by Geographic Information Systems (GIS) with ArcGIS Pro ESRI Software. Knowing the spatial patterns of the virus is essential if we are to contribute to effective policies and strategies [28], using spatial knowledge to design mitigate and control measures [29].

In this regard, the research proposes a method using 3D-bins and emerging hot spots to reveal space-time trends of the virus. Context variables are also explored, using the Ordinary Least Square (OLS) method to distinguish variables which are more closely correlated with virus incidence from demographic and territorial approaches. The methodological proposal can be adapted from the spatial and temporal viewpoints and helps to identify daily problem areas such as hot spots. It thus contributes to a culture of governance at regional level by revealing links and similarities between FUAs [30] for future waves and variants.

The case study looks at the regions of the Basque Country and Cantabria (northern Spain). In Spain, pandemic management was initially centralized at national level but was then decentralized to regional governments from the end of the lockdown in June 2020. Regions are thus the basic unit of pandemic management, coordinated at national level by the “Inter-territorial Committee”. The study area measures 12,555 km^2^, and has a population of 2,798,500 (Population register, 2021: *584,507 inhabitants in Cantabria and 2,213,993 in the Basque Country*). There are FUAs around the four main cities (Bilbao, Santander, Donostia-San Sebastián, and Vitoria-Gasteiz), which between them have 2,056,037 inhabitants (i.e., 73.5% of the population live in urban areas). The study period corresponds to the Omicron wave from 15th November 2021 to 31st March 2022, i.e., 136 days. Between them, the two regions recorded 1,603,096 confirmed COVID-19 cases in the 2 years from the beginning of the pandemic to the end date of the study period, of which 492,774 were recorded in the study period (about 4 months), i.e., 30.7% of the cases occurred in 18.8% of the time.

## Methodology

### Data

The research is based on the confirmed COVID-19 cases reported daily at municipal level by regional health authorities (Basque Government and Government of Cantabria). The study period corresponds to the sixth wave of infection in Spain: that of the Omicron variant. The cumulative total for the study area is 492,774, but the research considers the 491,816 cases where the municipality is known (91.8% of the total). The study brings together tabular data and a polygonal shape layer with 358 municipalities.

### Methods

A GIS project is implemented using ArcGIS Pro (ESRI GIS Company). The research workflow involves three stages, framed in geo-statistical and GIS cluster methods [31, 32].

The exploratory stage uses the Global Moran’s Index [33] to contrast the statistical significance of the distribution of COVID-19 cases aggregated at municipal level. The main stage analyzes 3D-bins and emerging hot spots based on Getis-Ord Gi* statistics [34] to identify hot spots as spreading areas and Mann-Kendall statistics to determine trends [35]. Following on from previous research based on 3D-bins implemented from geocoded microdata on COVID-19 cases [36–38], here the research considers the 3D-bins creation tool from previous locations (municipalities) as other authors have with point layers of cities [39] and polygons of counties and districts [40, 41]. 3D-bins based on municipalities accumulate cases over time in 14-day internal time slides, considering the 2-week periods commonly used by health authorities to calculate cumulative incidences. The methodology thus uses a relative parameter of time to avoid methodological distortions [42]. The workflow also includes a partial-time analysis from the beginning to the peak number of cases reported (5 January 2022) to study the spatial spread process in detail. Considering cumulative COVID-19 cases in each bin recorded over time, the emerging analysis provides a maximum of 17 pattern types (1 “no pattern detected”, 8 “cold spots”, and 8 “hot spots”). Hot spots are interpreted as spreading areas, cold spots are no-problem areas because of significant cold trends, and, finally, “no pattern detected” is essential to distinguish areas that have had cases but show no significant trend.

The third method is Ordinary Least Square (OLS) analysis. This method of generalized linear regression [43] seeks to analyze links between COVID-19 incidence and context variables. After considering nearly thirty demographic (structure indicators and density) and territorial variables (mainly accessibility), OLS reports three main statistics on each variable: coefficient and probability (to measure intensity and sign of correlation) and the variance inflation factor (VIF) (to avoid redundancies).

OLS testing contrasts stationarity with the Koenker index based on Breusch-Pagan (BP) [44]. If BP is significant (*p*<0.010 with a confidence level of 99%), the correlation is non-stationary. This is a key index because OLS is only methodologically appropriate when correlation is spatially homogeneous [45].

## Results

### The Wave with the Highest Peak

**Fig. 1:**
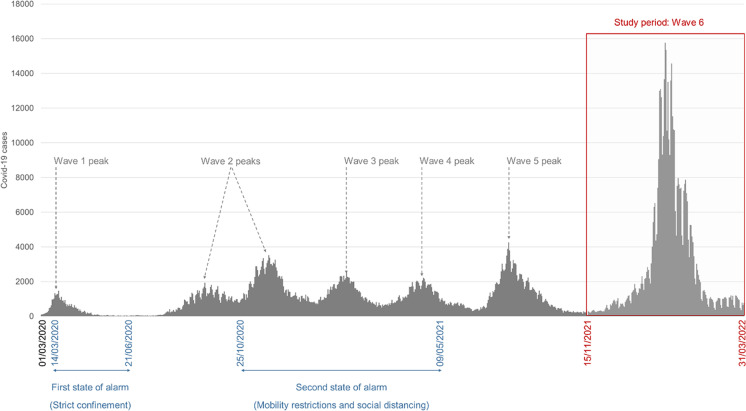
Daily trend in new confirmed COVID-19 cases in the study area from the beginning of the pandemic. Source: Regional Health Authorities. Basque Government and Government of Cantabria. Authors’ own work

The results focus on the biggest wave in terms of the number of cases (Fig. 1), with 492,774 confirmed cases (491,816 geocoded in municipalities) from November 2021 to March 2022, i.e., 30.7% of all cases since the beginning of the pandemic. These cases are mostly of the Omicron variant, the most transmissible encountered to date [27]. According to epidemiological situation reports from Spain’s Ministry of Health [46], the Omicron variant predominated with 91.1% of cases in Basque Country and 95.6% of cases in Cantabria in January 2022, decreasing to 76.2% and 68.3% respectively in March 2022.

### Non-randomness and Cluster Pattern of Cases

The Global Moran’s Index of the distribution of confirmed cases reports a significant clustered distribution at municipal level (*z*-score above 2.580, more precisely 3.159). There is a probability of less than 1% that the distribution of COVID-19 could be random considering municipal data.

**Fig. 2:**
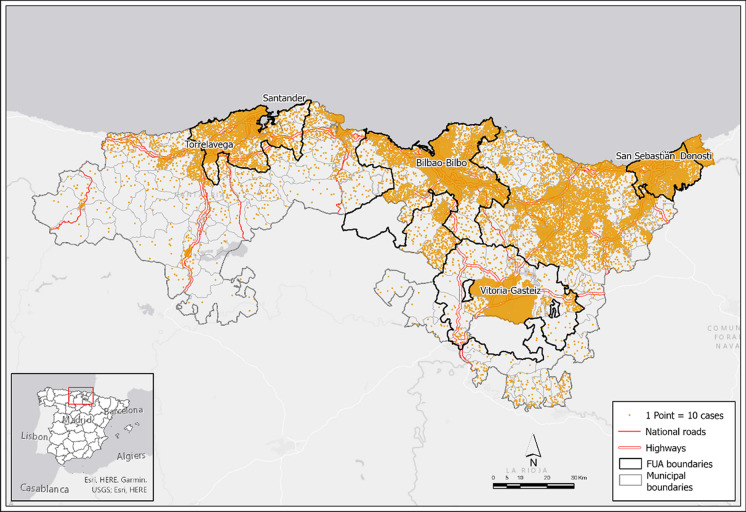
Density map of confirmed COVID-19 cases in municipalities (15 November 2021–30 March 2022). Source: Regional Health Authorities. Basque Government and Government of Cantabria. Authors’ own work

Cases are concentrated in the urban municipalities (Fig. 2) corresponding to the main cities (Bilbao, Santander, Donostia-San Sebastián, and Vitoria-Gasteiz) and their FUAs, identified at European level as dynamic supra-municipal units with high population concentration and intense commuting [47]. Additionally, municipalities connected by highways are highlighted for their concentration of cases. By contrast, inland rural municipalities —especially in Cantabria— show a more scattered distribution, except in rural service centers.

Despite the concentration of cases in FUA, with 70.9% of cases being found in 29.5% of the area (Table 1), there is no over-representation. The coefficient of location (CL) is below 1.0, which demonstrates that the municipalities in FUAs actually have fewer cases than would be expected in view of their volume of population. The only exception is the Donostia FUA, where the CL above 1.0, making it the area with the highest density (1031.8 inhab./km^2^).

**Table 1:** FUA sizes and distribution of confirmed COVID-19 cases

Areas	No Mun.	Cases	Population 2021	Area (sq.km)	Pop. Density	% Pop.	% Cases	% Area	CL
Bilbao FUA	54	178,225	1,040,873	1,258.8	826.9	37.2	36.2	10.0	0.97
Donostia FUA	13	67,306	342,038	331.5	1,031.8	12.2	13.7	2.6	1.12
Santander FUA	21	53,988	383,696	685.7	559.6	13.7	11.0	5.5	0.80
Vitoria-Gasteiz FUA	19	49,031	279,507	1,422.4	196.5	10.0	10.0	11.3	1.00
Total FUA	107	348,550	2,046,114	3,698.4	553.2	73.1	70.9	29.5	0.97
Outside FUA	251	143,266	752,386	8,858.0	84.9	26.9	29.1	70.5	1.08
Total area	358	491,816	2,798,500	12,556.4	222.9	100.0	100.0	100.0	-
FUA zones									
FUAs for main cities	4	164,402	959,821	414.8	2,313.9	34.3	33.4	3.3	0.97
Remaining FUAs	103	184,148	1,086,293	3,283.5	330.8	38.8	37.4	26.2	0.96

Regional Health Authorities. Basque Government and Government of Cantabria. National Institute of Statistics (Data from register of residents, 2021). Authors’ own work

### Space-Time Trends and the Role of FUAs in Spreading

**Fig. 3:**
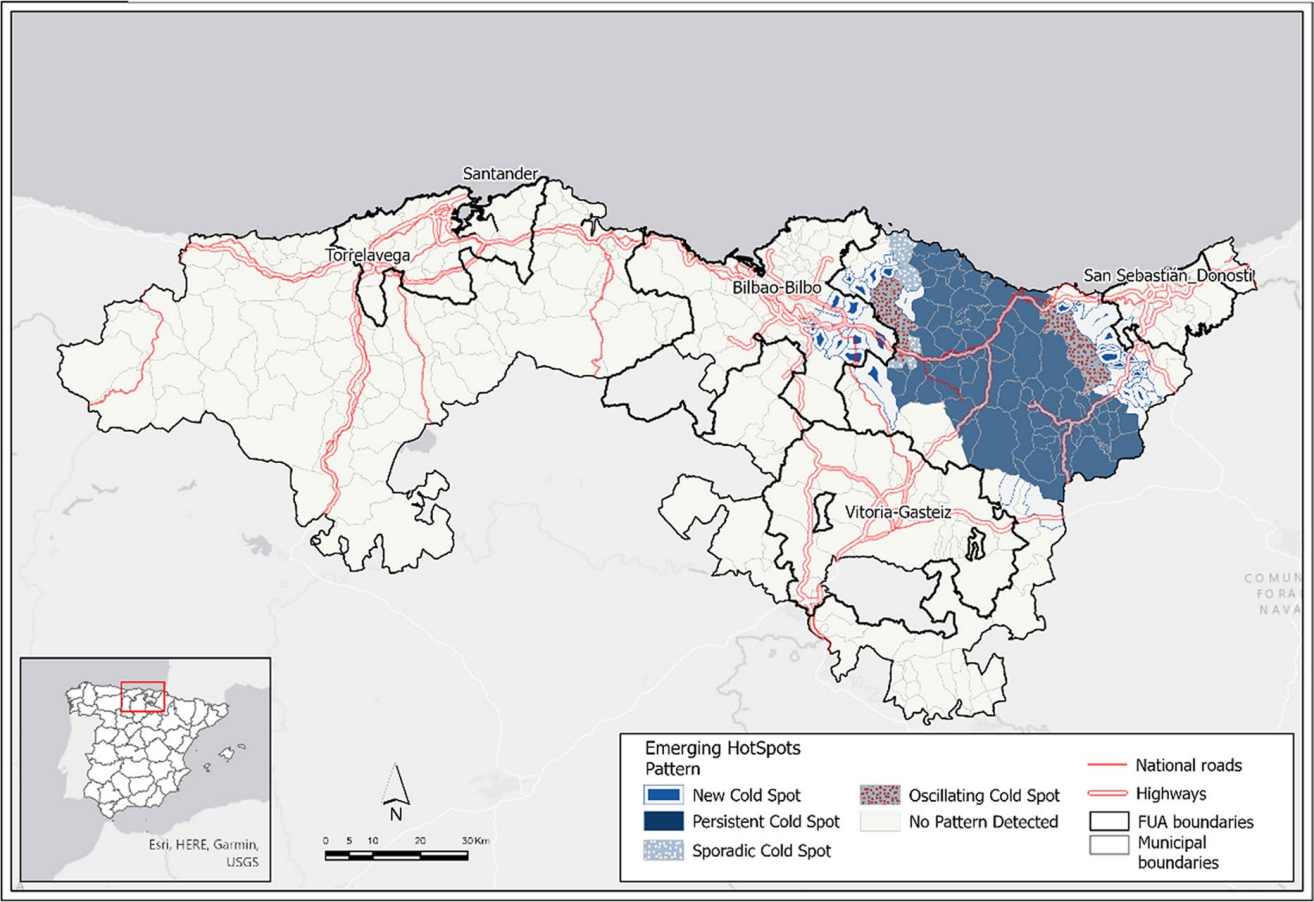
Emerging hot spots of the Omicron wave in the overall period from 15 November 2021 to 30 March 2022. Source: Regional Health Authorities. Basque Government and Government of Cantabria. Authors’ own work

**Fig. 4:**
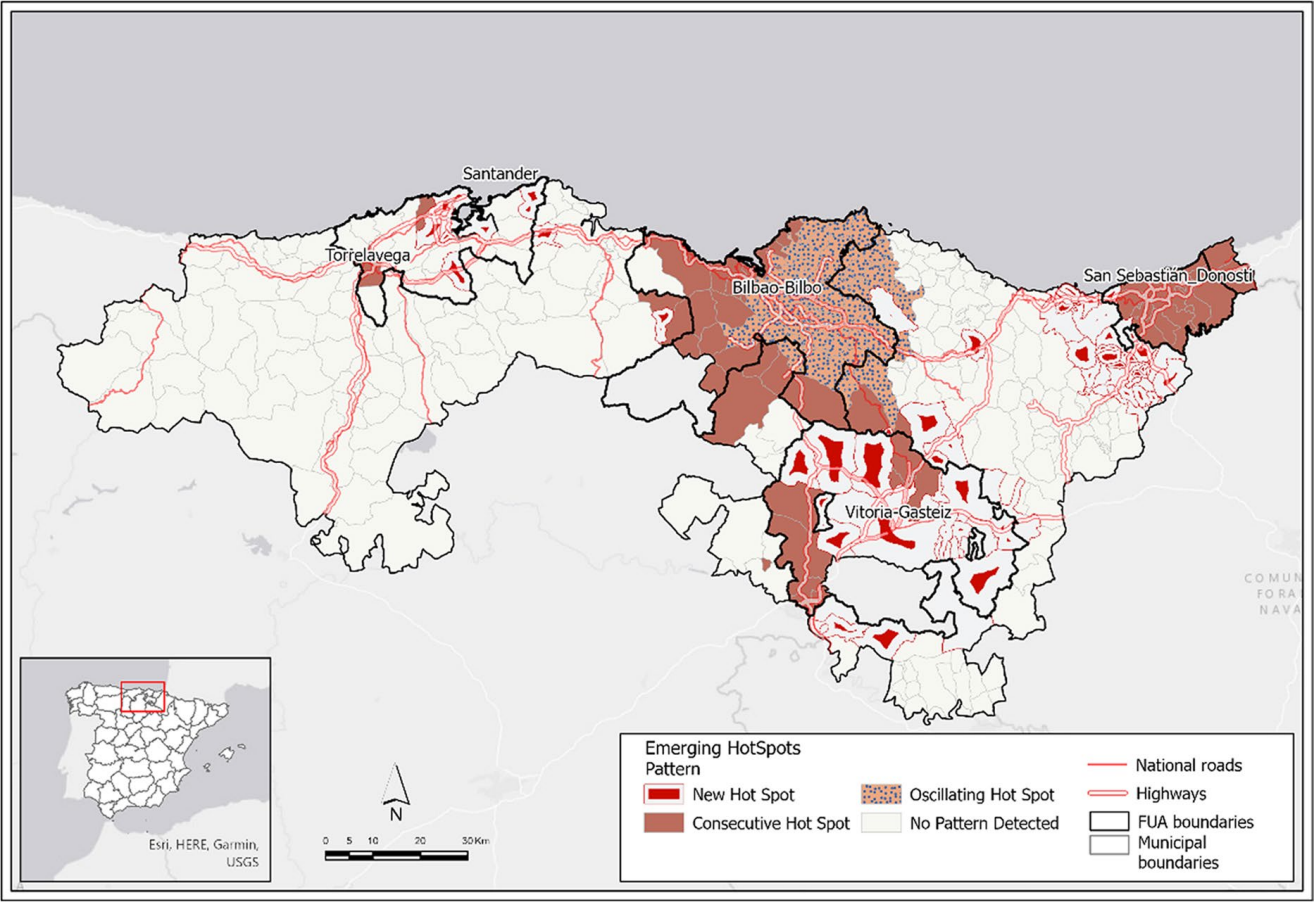
Emerging hot spots of the Omicron wave in the partial increasing period from 15 November 2021 to peak on 5 January 2022. Source: Regional Health Authorities. Basque Government and Government of Cantabria. Authors’ own work

The overall emerging hot spot analysis shows the end of the Omicron wave according to the leading non-significant pattern and the presence of cold patterns in some municipalities of the Basque Country, between the three Basque FUAs (Fig. 3). The absence of hot spots is another important finding that confirms the end of the spread in the period analyzed. Nevertheless, the partial emerging model from the beginning to the peak on 5 January 2022 reveals the role of FUAs as drivers for spreading with a broad, significant hot spot area from the Bilbao FUA to the Vitoria-Gasteiz FUA, another major persistent area in the Donostia FUA and new hot spots in the Santander FUA (Fig. 4). Here, two speeds are identified, first in the Basque Country FUA and then in the Santander FUA in Cantabria, with fewer hot spot municipalities, some of them with new patterns.

Most FUA municipalities are not significant in the overall period (94.1% of FUA area), but in the partial increasing period up to 5 January 2022, FUA municipalities show significant patterns (hot spots) in 85.0% of the FUA area, where 98.9% of FUA cases occur (Table 2). Therefore, in the increasing period of the wave, FUAs contribute decisively to the spread of the virus.

**Table 2:** Emerging patterns for the overall and partial periods

Overall period	Municipalities			Cases			Area		
15/11/2021–30/03/2022	Total	% Sig.	% Non-Sig.	Total	% Sig.	% Non-Sig.	Total	% Sig.	% Non-Sig.
FUA municipalities	107	8.4	91.6	348,550	4.7	95.3	3,698.4	5.9	94.1
Outside FUA	251	40.2	59.8	143,266	49.7	50.3	8,858.0	22.6	77.4
Total	358	-	-	491,816	-	-	12,556.4	-	-
Partial period	Municipalities			Cases			Area		
15/11/2021–5/01/2022	Total	% Sig.	% Non-Sig.	Total	% Sig.	% Non-Sig.	Total	% Sig.	% Non-Sig.
FUA municipalities	107	91.6	8.4	139,382	98.9	1.1	3,698.28	85.0	15.0
Outside FUA	251	38.6	61.4	64,468	48.9	51.1	8,857.96	29.4	70.6
Total	358	-	-	203,850	-	-	12,556.24	-	-

% Sig. indicates significant emerging patterns (cold or hot depending on the cases). By contrast, % Non-Sig. indicates “no pattern detected”.

Source: Regional Health Authorities. Basque Government and Government of Cantabria. Authors’ own work

**Fig. 5:**
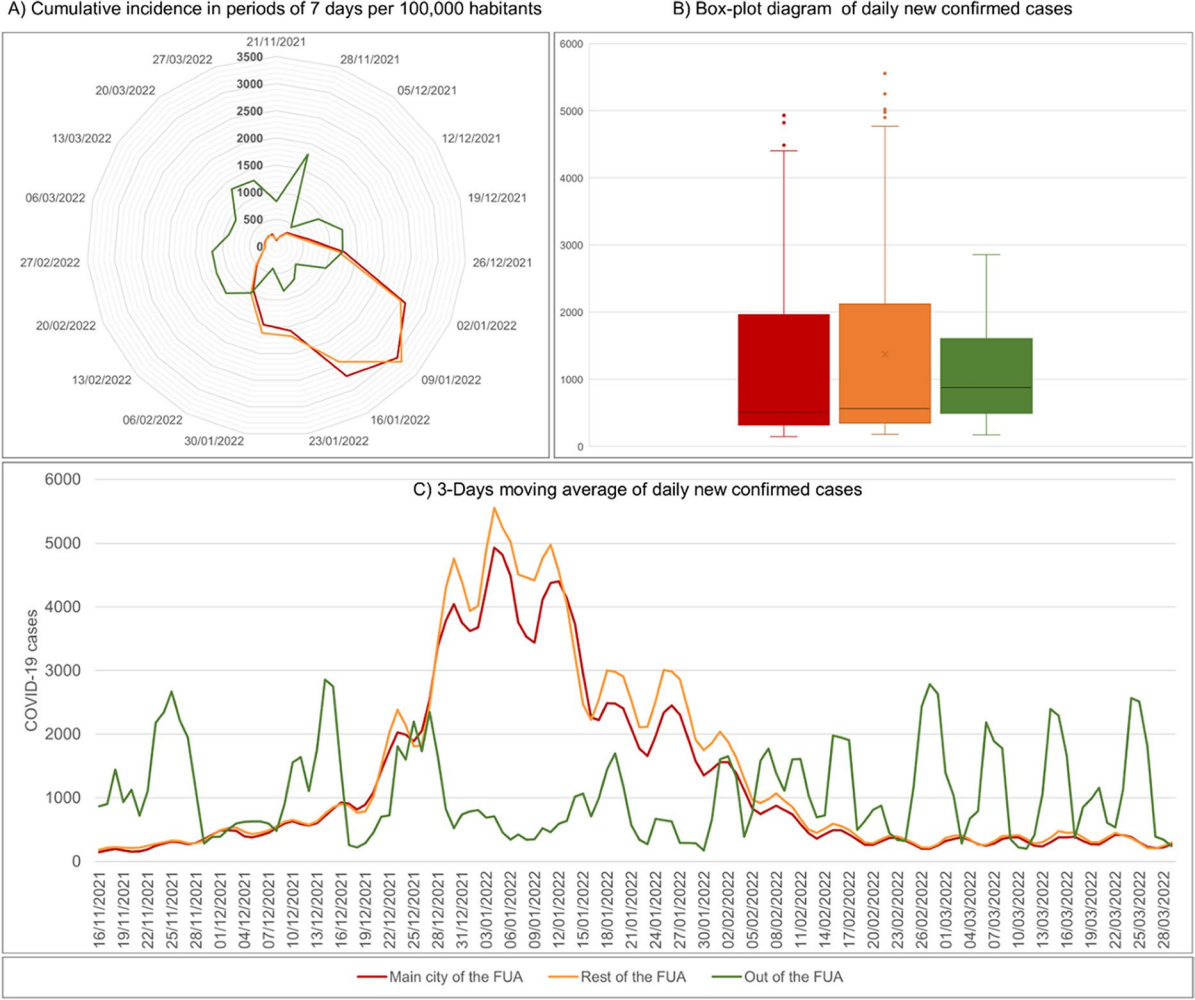
Evolution of COVID-19 cases per zone (15 November 2021–30 March 2022). Source: Regional Health Authorities. Basque Government and Government of Cantabria. Authors’ own work

The daily trends in cases are very different inside and outside FUA boundaries. Non-FUA municipalities show a slow, erratic trend in cumulative incidence over the whole period, while FUA areas show concentrated, high rates of spread of the virus in a short period of 1 month (Fig. 5A), with very fast increases from mid December 2021, a clear, broad peak of 2 weeks from the end of December 2021 to mid-January 2022 and then a progressive decrease (Fig. 5C). 5 February 2022 marks the turning point of the highest incidence of the virus in areas outside FUAs as a new stage in the evolution of the pandemic. Core FUA municipalities and the remaining peripheral FUA municipalities have very similar patterns, with a median of about 500 new cases per day and a third quartile of about 2000 new cases per day (Fig. 5B). Outside FUAs, the interquartile range is lower and the highest typical figure is under 3000, while in FUA municipalities, it rises to over 4000 in core municipalities and almost 5000 in the rest of the FUAs.

**Fig. 6:**
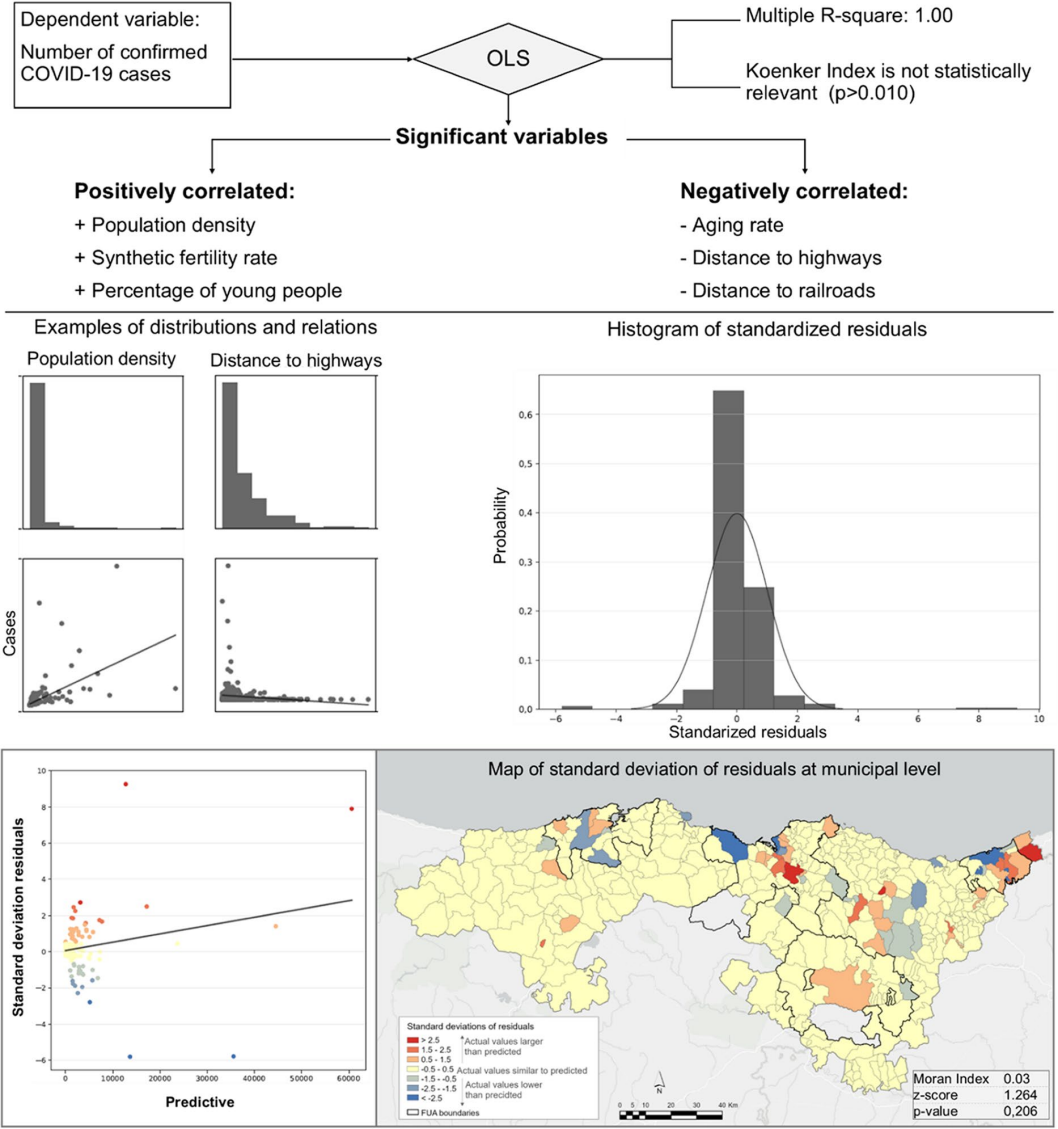
OLS report of context variables at municipal level. Source: Regional Health Authorities. Basque Government and Government of Cantabria. Authors’ own work

Finally, according to the exploratory analysis of context variables related to COVID-19 incidence, the OLS multiple R-square is 1.00, so the dependent variables considered explain 100% of COVID-19 cases in a linear regression (Fig. 6). Chi-square *p*<0.050 means that the model is statistically significant and the Koenker index of *p*>0.010 demonstrates that spatial relations are stationary. Therefore, the model is spatially uniform in our study area.

According to OLS results for coefficient, probability, and VIF, we obtain three variables which are positively correlated with COVID-19 incidence, linked to progressive areas (densely populated, young population, and more children per woman) and three negatively correlated variables, such as the aging rate, which again corroborates the role of demographic structure, and accessibility variables related to mobility (distance to highways and railroads).

Estimating a predictive model at municipal level lies beyond our research goals, but the OLS results are significant in that approach, according to the standard deviation residuals. As shown in Fig. 6, 249 municipalities (69.6% of the total) show standard deviations between −0.5 and +0.5 and the Global Moran Index shows a *z*-score of 1.264 and a *p* value of 0.206, so the spatial pattern of standard deviation residuals seems considerably random. This clearly supports the idea that the OLS model demonstrates the dispersion of residuals has no structure, although they are mainly in FUA areas and closest municipalities, so the model is correctly specified. FUA areas show municipalities with actual values larger than predicted (in red color) and in the outskirts appear some municipalities with actual values lower than predicted, as shown in the map of Fig. 6.

## Discussion

### Key Findings

The distribution of COVID-19 cases at municipal level is statistically significant according to the Global Moran’s Index, as other research has demonstrated using smaller areas such as zip codes [22] or even points for geocoded cases [36]. Confirmed cases are concentrated in urban areas and municipalities connected by highways. In fact, OLS analysis highlights distances to highways and railroads as negatively correlated with COVID-19 incidence, as found by other authors in analyzing commercial prosperity and accessibility at municipal level [48].

One interesting result is that COVID-19 cases are not over-dimensioned in urban areas, as demonstrated by CL under 1.0. So, the role of FUAs in the Omicron wave does not correspond to an over-concentration of cases. FUAs are important drivers of spread, as hot spot patterns demonstrate in the partial emerging analysis up to 5 January 2022. Studies of health in FUAs are often focused on pollution and green areas and their ecosystem services [49]. Therefore, we cannot contrast properly our results with other research about health spatial patterns on FUAs. Nevertheless, there is evidence about the role of urban scaling in health outcomes, not only size, but also proximity and mobility among urban areas, as FUAs [50]. Urban and metropolitan areas are essential in tackling the pandemic, and the spread in dynamic urban areas, such as FUAs, as key areas to analyze the spatial behavior of the virus at regional and local scale [51, 52]. In our results, FUAs have positive trends in the upward period of the wave, and the accumulation of positive cases is higher and shorter in time than in rural areas, where the pattern is mainly not statistically significant, and the daily trend in cases is erratic. As other authors state, urban areas are protagonist in the virus spread, having in consideration the concentration of population and cases, and other factors as crowding, spatial concurrence [24], mobility, and economic and demographic conditions [53]. Furthermore, our research confirms that highly transmissible variants such as Omicron and community transmission periods spread the virus to occupy medium-sized areas (such as rural service centers and inter-FUA municipalities) after the main cities such as Bilbao [24]. Cold spots and “no pattern detected” areas found in the global emerging model demonstrate that the end date of the study period corresponds to the end of the Omicron wave, with no hot spot patterns and a dominant model of cold trends.

Finally, data series of COVID-19 cases at municipal level are spatially stationary in relation to demographic and territorial variables. This contradicts the non-stationary behavior detected in other research at intra-urban scale with geocoded cases analyzed as points (not aggregated) [37]. Thus, municipal entities seem to be an adequate intermediate level between intra-urban and regional approaches to analyze correlations with context variables. Furthermore, many countries publish statistical data at municipal level (or similar). This means that the proposed methodology is exportable to other countries or study areas and re-scalable to other aggregation entities. Furthermore, this method monitors clustering during the evolution of the pandemic and detects in real time the location and type of problem areas such as hot spots, which is essential for pandemic response [54], especially in periods of in which people must live with the virus without strict lockdowns. Daily new cases are aggregated by area (municipalities, counties, etc.) and it can be included in the ArcGIS Pro project. Thus, new 3D-bins and emerging hot spots analysis will show problem areas in real time. Here, time parameter is the relative 2-week period and spatial parameter is based on aggregated areas. Additionally, geoprocessing model can be automated using Model Builder in ArcGIS Pro if health authorities need to monitor the impact of pandemic on population health periodically.

### Limitations

The research has some limitations. In regard to the variables analyzed, data on the proportion of people vaccinated are not available at municipal level, so the model does not include vaccine data, although some authors state that vaccination does not control the spread [26]. Some context variables, such as density, need to be improved. A clear correlation was obtained between COVID-19 incidence and density as an explanatory variable, but more advanced analysis requires “effective local density,” considering only residential areas in each municipality instead of the total area [55]. COVID-19 severity or mortality was not considered, although there is scientific evidence about the disparities in COVID-19 mortality due to social determinants of health [56, 57]. Deeper research will be necessary in the future.

There are also factors which limit the applicability and exportability of the proposed methodology. Many countries are reducing data monitoring and reporting of the pandemic. Two obstacles are identified: loss of temporal granularity and presence of data for certain collectives only (e.g., vulnerable people). This reduces the possibility of conducting continuous emerging analyses in future waves. On the other hand, the results are not easy to apply to pandemic management, due to a multi-tier administrative organization (regions, municipalities, and administrative health units, among others) [11] which makes coordination and governance harder for health authorities, where the municipal level is essential to adapt and converge HiAP and Health Cities approaches [8]. Furthermore, other interesting areas in Health Geography studies are Basic Health Areas (BHA). In urban areas, BHA are more disaggregated than municipalities; meanwhile in rural areas, BHA could be more aggregated in comparison to the municipal level. The benefit of BHA is that are the management health areas in Spanish regions and data of comorbidities can be obtained at that level. According to this, some authors state that is more adequate BHA than administrative boundaries, as counties or municipalities [58, 59]. In any case, the proposed methodology can be exported and applied to BHA at regional level.

## Conclusion

After 2 years, the COVID-19 pandemic continues to challenge health policies both globally and locally. Research focused on recent variants, such as Omicron, which is more transmissible than previous variants, reveals keys to spatial patterns for the design of effective policies. Urban areas (cities and outskirts) concentrate hot spots in the upward period of the Omicron wave. Subsequently, spatial spread affects medium-sized areas, rural service centers, and other municipalities. Therefore, fast action in the drivers of spread (functional urban areas) can help to prevent subsequent spread. Daily tracking of pandemic trends at municipal level using 3D-bins and emerging hot spot analysis is essential to identify municipalities with hot spot patterns and design fast control measures such as restrictions on mobility and gatherings, among others, or even large-scale prevention campaigns to detect asymptomatic cases or improve vaccination levels.

A multiscale approach is needed in the spatial analysis of the virus, and in that context, the use of GIS methods is essential. The methodology proposed here is adaptable and replicable in other case studies and using other boundaries, as interesting Basic Health Areas. Based on global principles of geo-prevention (health and safety areas) and the WHO HiAP philosophy, municipal diagnosis seems a suitable way of taking local decisions adapted to recognized boundaries. Taking municipalities as a level for decision-making in pandemic management helps with vertical and horizontal coordination, as was cited above state. Urban health governance requires strategic spatial reports such as those drawn up here, applying GIS location intelligence methods.
